# Thermal Management Approach to Stabilization of Disordered Active Sites for Sabatier Reaction

**DOI:** 10.1002/advs.202409048

**Published:** 2024-12-04

**Authors:** Delong Duan, Di Wu, Hongwei Shou, Chuansheng Hu, Canyu Hu, Min Zhou, Ran Long, Yingpu Bi, Yujie Xiong

**Affiliations:** ^1^ Hefei National Research Center for Physical Sciences at the Microscale Key Laboratory of Precision and Intelligent Chemistry School of Chemistry and Materials Science National Synchrotron Radiation Laboratory School of Nuclear Science and Technology University of Science and Technology of China Hefei Anhui 230026 China; ^2^ Suzhou Institute for Advanced Research University of Science and Technology of China Suzhou Jiangsu 215123 China; ^3^ State Key Laboratory for Oxo Synthesis and Selective Oxidation National Engineering Research Center for Fine Petrochemical Intermediates Lanzhou Institute of Chemical Physics Chinese Academy of Sciences Lanzhou Gansu 730000 China

**Keywords:** disordered active sites, heterogeneous catalysis, Sabatier reaction, Thermal management, thermochemistry

## Abstract

The transition metal nanocatalysts containing disordered active sites can potentially achieve efficient Sabatier reactions with high selectivity. However, it remains a challenge to maintain the stability of these active sites in such an exothermic reaction. Here, a thermal management approach is reported to address this challenge. Specifically, an efficient and stable catalytic system is developed by integrating urchin‐like Ru nanoparticles with disordered active sites (*d*‐RuNUs) and multi‐walled carbon nanotubes (MWCNTs) as heat transfer framework, which achieves a CH_4_ yield of 3.3 mol g^−1^ h^−1^ with nearly 100% selectivity in 12 h. The characterizations reveal that the thermal‐induced crystallization seriously weakens the adsorption of CO_2_, leading to significant degradation of catalytic performance. The heat transfer simulation confirms that the MWCNTs with high thermal conductivity play a key role in rapidly redistributing the reaction heat, thereby preventing the crystallization of disordered structures. This work elucidates the deactivation mechanism of disordered active sites in exothermic reactions and opens the avenue for local thermal management of non‐thermal equilibrium reactions.

## Graphical abstract

The (photo‐)thermal catalytic performance of urchin‐like Ru nanoparticles with disordered active sites (*d*‐RuNUs) degrades in the Sabatier reaction because of crystallization. Leveraging thermal management by multi‐walled carbon nanotubes (MWCNTs) with high thermal conductivity, the performance can be maintained through the stabilization of disordered active sites.

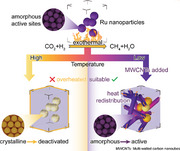

## 1 Introduction

The Sabatier reaction is a key technique for renewable energy storage in power‐to‐gas conversion as well as environmental control and life support systems (ECLSS) in manned spacecraft, which involves heterogeneous catalysis toward highly selective hydrogenation of carbon dioxide (CO_2_) to methane (CH_4_).^[^ ^1^ ^]^ Recently, transition metal‐based nanocatalysts have received much attention due to their high selectivity for CH_4_ by facilitating formate intermediate species.^[^ ^2^ ^]^ To further improve the performance and stability of the catalysts, enormous efforts have been devoted to optimizing the morphology, composition, and dispersion of active sites.^[^ ^3^ ^]^ In particular, it has been found that a high disorder degree in the catalyst structure, indicating the presence of more disordered active sites, can significantly contribute to realizing the ultrahigh performance of the (photo)thermal‐driven catalytic reactions.^[^ ^4^ ^]^ For instance, the amorphous/crystalline interface of Cu/amorphous‐ZrO_2_ provides suitable active sites that can greatly promote turnover frequencies and selectivity of methanol.^[^ ^5^ ^]^ However, these disordered catalysts often suffer from serious deactivation due to the thermodynamic instability of the amorphous structure,^[^ ^6^ ^]^ and localized thermal oscillations/accumulations during the reactions,^[^ ^7^ ^]^ which severely limits their further application in the exothermic Sabatier reaction (Δ_r_Hϴ m(298.15 K) = –252.9 kJ mol^−1^).^[^ ^8^ ^]^ For this reason, it is imperative to maneuver the heat transfer process of a specific catalytic system, preventing the catalyst deactivation caused by local heat accumulation.

Herein, we demonstrate a stabilization strategy for disordered active sites in the Sabatier reaction, using urchin‐like Ru nanoparticles with disordered active sites (*d*‐RuNUs) as a model catalyst. Such a model catalyst shows excellent activity in the initial Sabatier reaction with CH_4_ yield up to 2.8 mol g^−1^ h^−1^, but only 39.5% CH_4_ yield can be maintained after 12 h reaction. As deciphered by pair distribution function (PDF) profiles and X‐ray absorption fine structure (XAFS) spectra, the *d*‐RuNUs undergo an obvious thermal‐induced crystallization transition. The crystallization reduces CO_2_ adsorption capacity, which in turn induces the degradation of catalyst performance. Based on the results of heat transfer simulation, the addition of MWCNTs with high thermal conductivity can remarkably facilitate the redistribution of reaction heat. As a result, the thermal damage to the disordered active sites can be restrained, which allows the catalytic system to maintain a superior and stable CH_4_ production rate of 3.3 mol g^−1^ h^−1^ with nearly 100% selectivity in a 12 h test.

## 2 Results and Discussion

**Figure 1:**
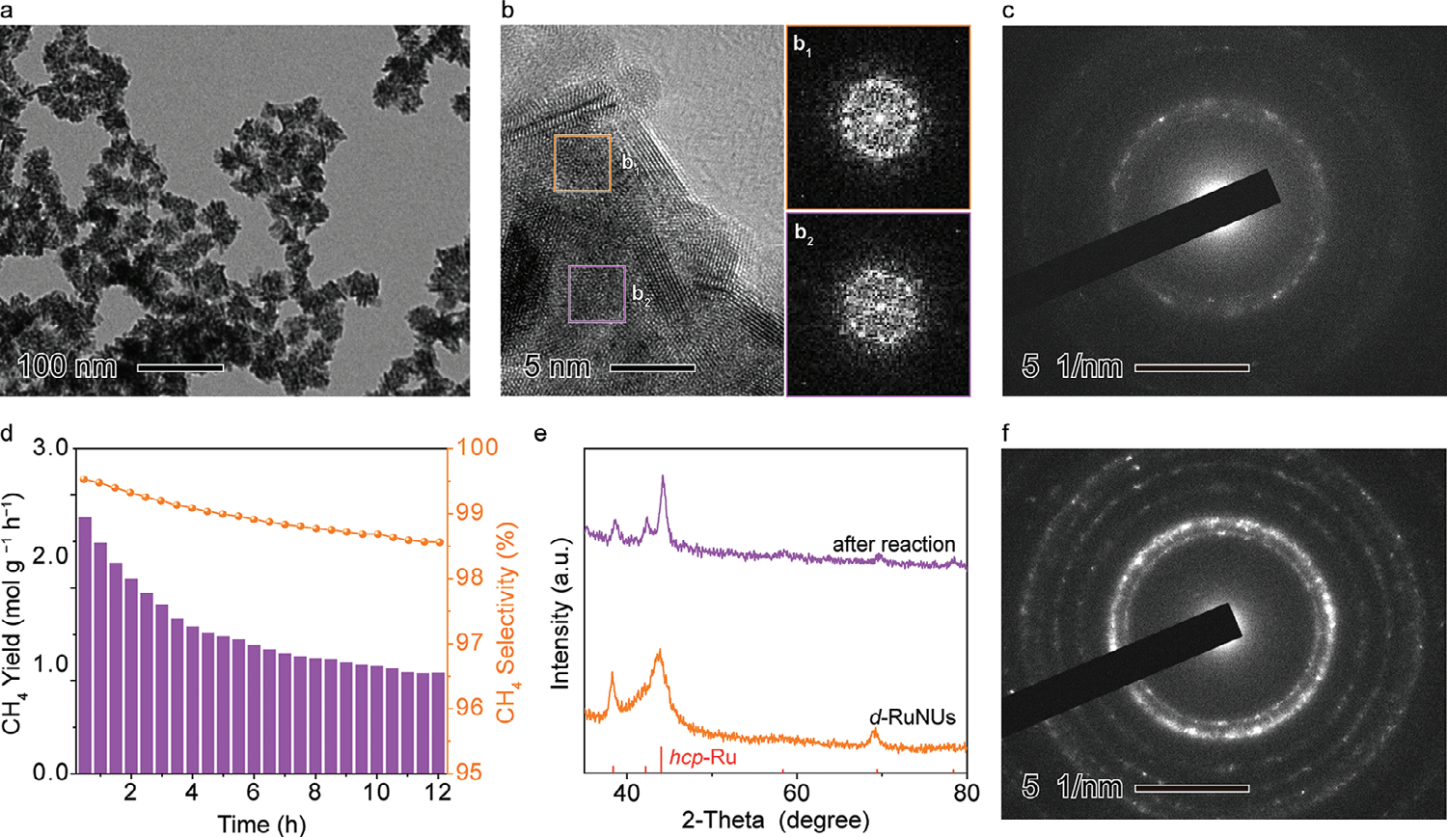
a) TEM image of freshly synthesized *d*‐RuNUs. b) HRTEM image of *d*‐RuNUs with b_1_ and b_2_ boxes marking disordered regions. b_1_ and b_2_) FFT patterns acquired from b_1_ and b_2_ boxes in (b), respectively. c) SAED pattern of fresh *d*‐RuNUs. d) Thermal catalytic Sabatier reaction performance of *d*‐RuNUs (573 K, GHSV = 450000 mL g^−1^ h^−1^). e) XRD patterns of fresh *d*‐RuNUs and the catalyst after the reaction. f) SAED pattern of *d*‐RuNUs after the reaction.

The *d*‐RuNUs were synthesized via a simple alcohol reduction process with poly(vinyl pyrrolidone) (PVP) as a stabilizer and RuCl_3_·*x*H_2_O as a metal precursor. Transmission electron microscopy reveals that *d*‐RuNUs are well dispersed and have an urchin‐like morphology with an average size of 38.1 nm (**Figure** **1**a; FigureS1, Supporting Information). High‐resolution TEM (HRTEM) further illustrates that the *d*‐RuNUs obviously contain disordered regions (see box b_1_ and b_2_, Figure 1b), which can be confirmed by the diffuse rings in corresponding Fourier transform (FFT) patterns. In addition, the selected area electron diffraction (SAED) pattern with ambiguous diffraction spots and thick diffraction rings in Figure 1c also indicates the poor crystallinity of *d*‐RuNUs.

To evaluate the catalytic Sabatier reaction performance of *d*‐RuNUs, the reaction was conducted at different temperatures. Driving by the heat, the initial yield of CH_4_ increases with the rising temperature (Figure S2, Supporting Information). However, the obvious decrease in performance was observed when the reaction temperature was above 600 K. At the optimal reaction temperature of 573 K, the yield of CH_4_ could reach 2.8 mol g^−1^ h^−1^ at the beginning but then decreased dramatically to 1.1 mol g^−1^ h^−1^ after 12 h reaction (Figure 1d). In addition, as indicated by the ^13^CO_2_ isotope labeling experiment result (Figure S3, Supporting Information), the detected ^13^CH_4_ and ^13^CO identify the fact of ^13^CO_2_ as the carbon source.

Considering that the coke formation is one of the common factors for catalyst deactivation in thermal‐driven CO_2_ conversion,^[^ ^9^ ^]^ we first attempted to regenerate the used *d*‐RuNUs in a flowing H_2_O/Ar.^[^ ^10^ ^]^ However, an even lower CH_4_ yield was obtained after the treatment (Figure S4, Supporting Information). Meanwhile, the thermogravimetric analysis (TGA) data also show no observable weight loss due to the potential coke removal (Figure S5, Supporting Information).^[^ ^11^ ^]^ C K edge X‐ray absorption (XAS) spectroscopy was further employed to analyze the C species on the used *d*‐RuNUs. As shown in Figure S6a (Supporting Information), the two obvious peaks located at 285.6 and 288.9 eV are assigned to π^*^ transitions of the C═C and C═O group, respectively,^[^ ^12^ ^]^ while the broad peak ≈292.5 eV can be attributed to the σ^*^ transition of residual capping reagent or solvent during the synthesis process.^[^ ^13^ ^]^ Obviously, the intensity of π^*^(C═C) and σ^*^ transition both decreased after the reaction, implying a lowered surface C species concentration.^[^ ^14^ ^]^ The Raman spectra of the fresh and used catalyst samples also show barely detectable characteristic D‐band (1300 cm^−1^) and G‐band (1580 cm^−1^) of amorphous carbon species (Figure S6b, Supporting Information),^[^ ^15^ ^]^ excluding the possibility of coke formation.

It is known that the size of nanoparticles is one of the key features that affect the activity.^[^ ^16^ ^]^ Whereas, the TEM images and particle size distribution data of fresh and after reaction *d*‐RuNUs depicted no size or morphology change (Figure S7, Supporting Information). We then looked into the evolution of catalytic sites. In the Ru XAS spectra (Figure S8, Supporting Information), the two peaks at 464.2 and 486.6 eV can be assigned to the M_3_ and M_2_ edges of Ru, respectively. However, no XAS peak shift or intensity change occurred after the reaction, indicating the invariant electronic structure and oxidation state of *d*‐RuNUs. The same result was also approved by Ru 3p XPS spectra (Figure S9, Supporting Information). Meanwhile, X‐ray diffraction (XRD) characterization was performed to resolve the changes in the crystal structure (see Figure 1e), which is another key parameter to determining catalyst activity. Interestingly, the sharpened XRD patterns demonstrated an increased crystallinity of *d*‐RuNUs after the catalytic reaction. This result was also confirmed by the enhanced diffraction spots and rings in the SAED pattern of used *d*‐RuNUs (Figure 1f).

**Figure 2:**
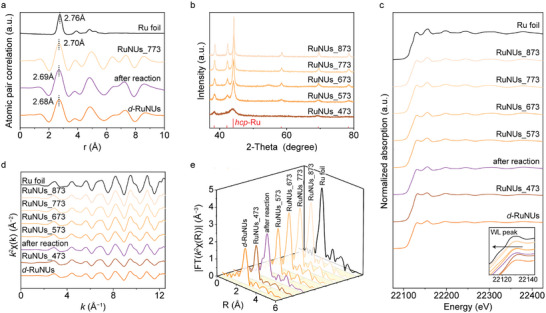
a) PDF profiles, b) XRD patterns, c) XANES, partial EXAFS and enlarged XANES spectra, d) *k* ^2^‐weighted EXAFS oscillation (k‐space), and e) *k* ^2^‐weighted FT‐EXAFS spectra of fresh *d*‐RuNUs, *d*‐RuNUs after reaction and RuNUs_*x* (x represents the annealing temperature) at the Ru K edge.

In order to determine the cause of *d*‐RuNUs crystallization, we prepared the samples by annealing *d*‐RuNUs under different temperatures (RuNUs_*x*, *x* represents the annealing temperature in Kelvin) to regulate the crystallinity. Here, the Ar atmosphere was employed as a shielding gas to minimize the chemical changes of the catalyst during thermal treatment, whose feasibility was further validated by the identical XRD patterns of the samples annealing in Ar and H_2_, respectively (Figure S10, Supporting Information). The SAED pattern of RuNUs_473 shows a similar disordered feature to fresh *d*‐RuNUs, while RuNUs_773 was significantly crystallized (see Figure 1c; Figure S11, Supporting Information). Furthermore, **Figure** **2**a illustrates the PDF profiles of the nearest coordination shells. Notably, the peak positions of the first coordination shell of the used *d*‐RuNUs and RuNUs_773 were both shifted to the longer radial distance, thus closer to the Ru foil with the longest one, implying a decrease in the disorder degree of these samples.^[^ ^17^ ^]^ Moreover, as demonstrated by the XRD patterns in Figure 2b, the diffraction peaks of RuNUs were narrowed with increasing annealing temperature, which is consistent with the results in Figure 1e. It is worth noting that the peaks of all samples are assigned to the hexagonal close‐packed (*hcp*) phase of Ru (JCPDS No. 70–0274) and no other phase can be found. As such, it is reasonable to infer that the thermal‐induced recrystallization of the disordered structure is responsible for the deactivation of the catalyst.

Synchrotron radiation‐based XAFS spectroscopy was further employed to monitor the relationship between disorder degree and annealing temperature owing to its sensitivity toward atomic dislocation.^[^ ^18^ ^]^ As shown in Figure 2c and X‐ray absorption near edge structure (XANES) spectra depict that the white‐line (WL) peak of fresh *d*‐RuNUs is the highest among the samples. Given that the atomic disorder in crystals can raise the energy of the core‐level electron shell,^[^ ^19^ ^]^ the shift of WL peaks toward lower energy demonstrates the reduction of atomic disorder degree in the used and annealed samples. More importantly, the oscillation amplitude of the *k* ^2^‐weighted extended XAFS (EXAFS) in k‐space is a direct indicator of the atomic disorder degree due to its sensitivity to the variance of interatomic distance.^[^ ^20^ ^]^ As shown in Figure 2d, an increased amplitude resulting from annealing or reaction indicates a more uniform spacing of Ru atoms with a lower disorder degree.^[^ ^21^ ^]^ Moreover, the *k* ^2^‐weighted Fourier‐transform EXAFS spectra and fitting data were obtained to reveal the local coordination configuration of Ru. As shown in Figure 2e, the stronger intensities of Ru−Ru coordination peaks at 2.35 Å and larger coordination numbers (Figure S12 and Table S1, Supporting Information), which are closer to the features of a close‐packed hexagonal lattice of standard Ru foil, indicate a crystallization of disordered phases after annealing or reaction.

**Figure 3:**
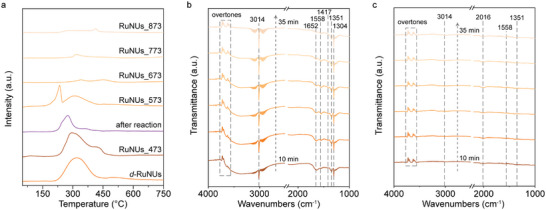
a) CO2‐TPD of fresh *d*‐RuNUs, *d*‐RuNUs after reaction and RuNUs_*x*. In situ DRIFTS spectra of b) fresh *d*‐RuNUs and c) RuNUs_873 (573 K, 80% H_2_/20% CO_2_).

Upon having a comprehensive understanding of the structure of *d*‐RuNUs, we further investigated the role of disorder degree in catalytic Sabatier reaction. The CO_2_ temperature‐programmed desorption (CO_2_‐TPD) experiment was conducted to quantify the adsorption capacity of CO_2_. As shown in **Figure** **3**a, the fresh *d*‐RuNUs have the largest desorption peak area, representing the highest amount of CO_2_ adsorption. However, the desorption peak areas of the used and annealed samples are reduced rapidly, illustrating the significant positive correlation between the CO_2_ adsorption ability and the disorder degree of *d*‐RuNUs.

To further gain insight into the influence of disorder degree on the subsequent process of CO_2_ catalytic activation, the in situ diffuse reflectance infrared Fourier‐transform spectroscopy (DRIFTS) was employed. In Figure 3b, The DRIFTS spectra of fresh *d*‐RuNUs were first acquired. The two broad peaks located at 3716 and 3610 cm^−1^ can be attributed to characteristic overtones of CO_2_,^[^ ^22^ ^]^ whose intensities remain almost unchanged, implying the partial pressure of CO_2_ is kept stable. The symmetric and asymmetric stretching vibration of COO at 1351 and 1558 cm^−1^ originate from the chemisorption of CO_2_ on the surface.^[^ ^23^ ^]^ Meanwhile, the weak peak located at 1417 cm^−1^ can be assigned to the adsorbed COOH, the common intermediate in CO_2_ hydrogenation. In the meanwhile, the intense peaks at 3014 and 1304 cm^−1^ are ascribed to the stretching vibration of C─H and the deformation vibration from CH_4_ product, indicating the high activity of *d*‐RuNUs.^[^ ^24^ ^]^ In contrast, the DRIFTS spectra of RuNUs_873 in Figure 3c only show weak signals of chemisorbed CO_2_ at 1351 and 1558 cm^−1^, which is consistent with the CO_2_‐TPD results. Notably, the relatively weak CH_4_ and the newly emerging CO signals at 3014 and 2016 cm^−1^ are due to the low CH_4_ yield and selectivity of RuNUs_873 (Figure S13, Supporting Information). It is now evident that the disorder degree of the active sites can effectively impact the abundance of adsorbed CO_2_ molecules and intermediate species, ultimately altering the catalytic performance. Therefore, it is easy to understand why the RuNUs_673, RuNUs_773, and RuNUs_873 with less disordered active sites exhibit a dramatic decline in their Sabatier performance (Figure S14, Supporting Information).

**Figure 4:**
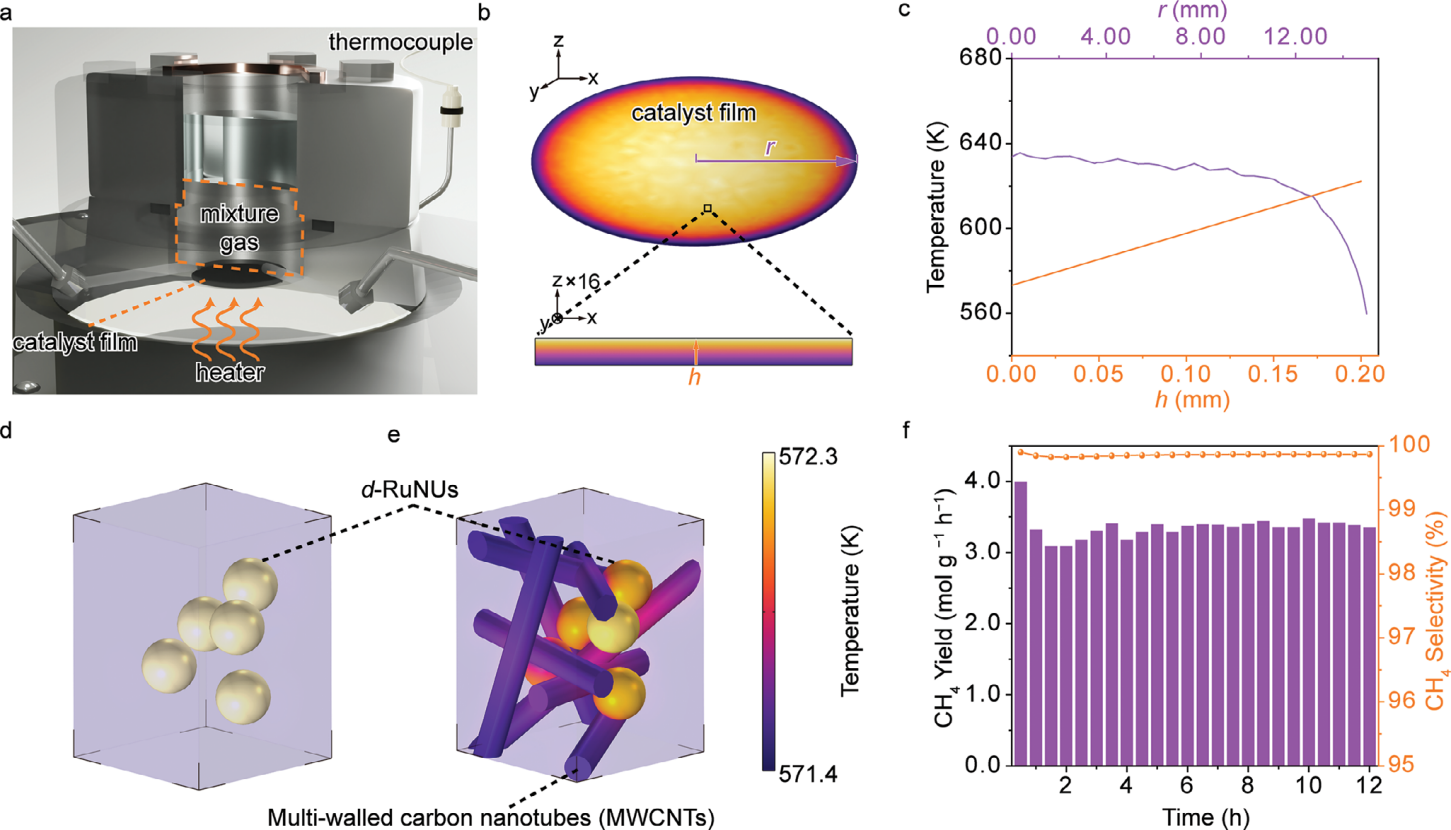
a) Cross‐section of photothermal catalysis system. b) Simulation of catalyst film with the illustration of radial direction vector (*r*) and thickness direction vector (*h*). c) Temperature distribution along *r* and *h* vectors acquired by heat transfer simulation. Surface temperature distribution of d) *d*‐RuNUs and e) *d*‐RuNUs/MWCNTs microzones acquired by heat transfer simulation. f) Thermal catalytic Sabatier reaction performance of *d*‐RuNUs/MWCNTs (573 K, GHSV = 450000 mL g^−1^ h^−1^).

After recognizing the indispensable role of the disorder degree, it is imperative to develop an approach to prevent the crystallization transition of the disordered active sites. However, the basic temperature control of the gas‐solid reactor has a very limited effect (see Figure S2, Supporting Information). To address this issue, we performed systematical heat transfer simulations to explore the thermal field characteristics of the microscopic catalyst structures. As shown in **Figure** **4**a, the *d*‐RuNUs are evenly distributed on a glass fiber film, which is positioned directly above a controllable heater. The space above the catalyst film is filled with a mixture of CO_2_ and H_2_ feed gases. In addition, the temperature on the underside of the catalyst film is kept at 573.0 K by controlling the heater power.

In order to investigate the thermal field distribution of catalyst film, we performed the simulation along the vector *r* with the radial (radius of 15 mm) direction and along vector *h* with the thickness (0.2 mm) direction from the bottom to the top (Figure 4b,c). Since the upper side of the catalyst film has more complete contact with the reacting gas, the accumulation of the reaction heat is more pronounced, resulting in a gradual bottom‐up temperature increase to 622.2 K, far exceeding the target 573.0 K. Furthermore, the thermal environment surrounding the catalyst nanoparticles was also thoroughly investigated by simulating a randomly selected microzone of the catalyst film. To simplify the model, we represented five *d*‐RuNUs as randomly distributed spheres. As shown in the temperature contour (Figure 4d), the maximum temperature regions are located on the surfaces of the *d*‐RuNUs, whose temperature is much higher than the surrounding environment. Such non‐uniformity of the thermal field distribution caused by the considerable reaction heat may eventually lead to the crystallization and deactivation of the *d*‐RuNUs due to local overtemperature.

To address such an issue, we introduced MWCNTs, a material with high stability under reaction conditions and high thermal conductivity to create high‐flux heat transfer channels in the catalytic system (see Figures S15–S17, Supporting Information). The simulation results show that the upperside temperature of the catalyst film drops significantly to 593.88 K (Figure S18, Supporting Information). Moreover, the result of the 1.5 ps simulation in Figure 4e demonstrates that the heat on the *d*‐RuNUs surface can be rapidly dissipated through MWCNTs. This suggests that the addition of MWCNTs can effectively redistribute the thermal field, thereby maintaining a suitable temperature for the catalysts and avoiding their thermal‐induced crystallization transition. As a result, the *d*‐RuNUs/MWCNTs achieve a stable CH_4_ yield of 3.3 mol g^−1^ h^−1^ with almost 100% selectivity in a 12 h test (Figure 4f).

Additionally, the *d*‐RuNUs also exhibit outstanding light‐absorbing properties (Figure S19, Supporting Information), enabling efficient photothermal‐driven Sabatier reactions (Figures S20–S22, Supporting Information). However, the incident light intensifies the nonuniformity of the energy input, resulting in a more significant degradation of the catalytic performance of *d*‐RuNUs, which can be verified by the elaborate in situ characterizations (see detail in Figures S23–S25, Supporting Information). In such a complex system, by constructing a catalyst film with efficient thermal conductivity, we can still effectively manage the heat distribution in the micro/nanostructures and achieve efficient and stable Sabatier reaction performance (Figure S26, Supporting Information). It should be noted that when the recrystallization of the catalyst is inevitable due to the overheated overall system temperature, thermal management can only partially suppress the performance decline (Figure S27, Supporting Information). Nevertheless, thermal management is still necessary; otherwise, a much lower system temperature is typically required to ensure the stability of the catalyst, which often leads to an unacceptably low reaction rate (see Figure S2, Supporting Information).

## 3 Conclusion

In summary, we have developed the *d*‐RuNUs catalysts with disordered active sites for efficient selective reduction of CO_2_. Comprehensive quasi‐in situ characterizations have proven that the thermal‐induced crystallization of catalyst nanoparticles during the reaction reduces the disorder degree of the crystal structure. The molecular behavior in the reaction indicates that the dramatic reduction in the CO_2_ adsorption capacity and the change in the activation pathway are the primary reasons for the degradation of the catalytic performance. By adding MWCNTs to construct a high‐flux heat transfer channel, the accumulated reaction heat on the catalyst surface can be rapidly redistributed. Thereafter, the crystallization transition of the disordered active site is inhibited to achieve an efficient and stable catalytic reaction system for the Sabatier reaction. This work clarifies the deactivation mechanism of disordered catalysts and emphasizes the significance of thermal management of the reaction microenvironment especially in exothermic reactions.

## Conflict of Interest

The authors declare no conflict of interest.

## Supporting information



Supporting Information

## Data Availability

The data that support the findings of this study are available from the corresponding author upon reasonable request.
