# Supplementary material for: Thermal Management Approach to Stabilization of Disordered Active Sites for Sabatier Reaction
Source: Adv Sci (Weinh). 2024 Dec 4;12(4):2409048. doi: 10.1002/advs.202409048 (PMC11775512; doi:10.1002/advs.202409048)
Supplement: Supplementary file 1 — Supporting Information [file ADVS-12-2409048-s001.pdf]

ADVANCED SCIENCE
Open Access

Supporting Information

for *Adv. Sci.*, DOI 10.1002/advs.202409048

Thermal Management Approach to Stabilization of Disordered Active Sites for Sabatier Reaction

Delong Duan, Di Wu, Hongwei Shou, Chuansheng Hu, Canyu Hu, Min Zhou*, Ran Long*, Yingpu Bi and Yujie Xiong**

Supporting Information
©Wiley-VCH 2021
69451 Weinheim, Germany

Thermal Management Approach to Stabilization of Disordered Active Sites for Sabatier Reaction

Delong Duan,+[a, b] Di Wu,+[a] Hongwei Shou,[a] Chuansheng Hu,[a] Canyu Hu,*[a] Min Zhou,*[a] Ran Long*[a] Yingpu Bi,[c] and Yujie Xiong*[a, b]

+ These authors contributed equally to this work.

DOI: 10.1002/anie.2021XX

Experimental Procedures

Chemicals

Diethylene glycol ($C_4H_{10}O_3$, DEG, 99.0%) and acetone (C_3H_6O, 99.5%) were purchased from Sinopharm Chemical Reagent Co., Ltd. Polyvinyl pyrrolidone (PVP, average M_W = 50000) was obtained from Sigm*a*-Aldrich. Ruthenium(III) chloride hydrate ($RuCl_3 \cdot xH_2O$, 35.0−42.0% Ru basis) was obtained from Aladdin. The water used in all experiments was deionized (DI). All the chemical reagents were used without further purification.

Preparation of fresh *d*-RuNUs, *d*-RuNUs annealed in Ar atmosphere and *d*-RuNUs after reaction

Typically, the urchin-like Ru nanoparticles (*d*-RuNUs) were prepared through simple thermal reduction in polyhydric alcohols. At first, 40 mL DEG solution containing 120 mg polyvinylpyrrolidone (PVP) was heated to 498 K and maintained in a three-neck flask. Then 4 mL DEG solution containing 60 mg $RuCl_3 \bullet xH_2O$ was injected into the flask by syringe pump at the rate of 0.3 mL/min. During the injection process, the solution turned from light brown to deep dark, and precipitates began to be generated. After refluxing at 498 K for 15 min, the solution was cooled to room temperature naturally. Second, the precipitates within the solution were separated by centrifuging and washed with acetone and DI water several times. Finally, the obtained urchin-like Ru nanoparticles with disordered active sites (*d*-RuNUs) were preserved in DI water for further use.

To synthesize RuNUs_*x* (*x* represents the calcination temperature in Kelvin) under different temperatures in Argon (Ar) atmosphere, *d*-RuNUs powder was placed in the tube furnace with an Ar gas flowing rate of 50 mL min^{-1}. After 90 min to remove air, the furnace was programmed to maintain at 473 K, 573 K, 673 K, 773 K, and 873 K for 4 h with a heating rate of 5 K min^{-1}, respectively. Finally, RuNUs_473 K, RuNUs_573 K, RuNUs_673 K, RuNUs_773 K, and RuNUs_873 K samples were obtained after cooling to room temperature spontaneously.

To prepare used *d*-RuNUs, the DI water-dispersed fresh *d*-RuNUs (~10 mg) were dropped on the heat-resistant FTO glass slide and let dry naturally. Then, the FTO glass slide was placed in the photothermal catalytic reactor with continuous CO_2/H_2 flowing at rates of 12 and 48 mL min^{-1}. After the reaction, the sample was obtained by scraping off the FTO glass slide after the system cooled to room temperature.

Characterizations

Powder XRD patterns were recorded by the Japan Rigaku rotation anode X-ray diffractometer equipped with a graphite monochromatized Cu Kα X-ray generator (λ = 1.54178 Å). TEM images were taken on a Hitachi Model H-7700 microscope at 100 kV. HRTEM images were taken on a JEOL JEM-2100Plus Single crystal cathode high-resolution transmission electron microscope at 200 kV. SAED patterns were acquired from a JEOL JEM-2100Plus single crystal cathode high-resolution transmission electron microscope at 200 kV. Visible-near-infrared absorption was tested on Shimadzu SolidSpec-3700. The Raman measurements were performed using a 473, 532, 633, or 785 nm laser with the power of 14.8 mW and an integration time of 10 s on a WITec alpha 300R confocal Raman microscope equipped with a CCD camera (UHTS 300 SMFC VIS-NIR, WITec). The in-situ temperature surveillance was conducted by a KAIPUSEN needle-type thermocouple that contacted with the catalyst film by Hasuncast SG7650 silicone thermal grease, of which the data was collected with the interval of 2 s in the first 60 s, and 300 s when temperature reached constant. Light source aging data was obtained on the interval of 0.5 h by measuring at a fixed position where the light intensity was 2.0 W cm^{-2} at the beginning.

Catalytic Sabatier reaction performance measurement

The photothermal- and thermal-driven catalytic Sabatier reaction was conducted in a photothermal reactor (NSVP50, Anhui Kemi Instrument Co., LTD.) with a volume of 50 mL. Typically, 2~8 mg of *d*-RuNUs or 2~8 mg multi-walled carbon nanotubes (MWCNTs) mixed with 2~8 mg *d*-RuNUs pervaded glass fiber film (*d*-RuNUs on GFF, *d*-RuNUs/MWCNTs on GFF) was placed in the reactor. The reactor was sealed except for inlet and outlet. Next, premixed high-purity (99.999%) 80% H_2/20% CO_2 feed gas at certain flowing rates was kept pumping in and out for 30 min to remove air. Before reaction, the sample was kept in dark for 30 min to adsorb CO_2 and H_2.For photothermal catalytic test, the catalyst was irradiated by a 300 W Xe-lamp (PLSSXE300, Perfect Light) at intensities of 1.5~3.0 W cm^{-2}; For thermal catalytic test, the built-in electric heating function of the reactor was set to maintained at temperatures of 523~648 K for 12 h. The product was analyzed by a gas chromatograph (GC, GC-2014, Ar and N_2 carrier, Shimadzu) continuously throughout the reaction period.

The yield of CH4(r_{CH_4}), CO(r_{CO}) and selectivity of CH4(S_{CH_4}) were calculated respectively as following Equations S1–S3:

$$r_{CH_4}(\mathrm{mol}\ g\ h^{-1}) = \frac{V_t \times [CH_4]}{V_m \times m_{cat}}$$

$$r_{CO}(\mathrm{mol}\ g\ h^{-1}) = \frac{V_t \times [CO]}{V_m \times m_{cat}}$$

$$S_{CH_4}(\%) = \frac{r_{CH_4}}{r_{CH_4} + r_{CO}}$$

where the V_t is the volume of feed gas that pass through the reactor per hour, V_m is the standard molar volume of gas, and the $[CH_4]$ and $[CO]$ are the concentrations of CH_4 and CO in off gas, respectively. The m_{cat} is the mass of catalyst.

Isotope-labelling Sabatier reaction experiment

The isotope-labelling experiments were performed by using pure $^{13}CO_2$ and $^1H_2$ as feeding gas. The gases were mixed to 1:4 ratio in a homemade 36.5 mL quartz tube that sealed *d*-RuNUs on GFF. Then, the photothermal catalytic Sabatier reaction was performed for 5 min. The $^{13}CH_4$ and ^{13}CO products were analyzed by GC−MS (7890 A and 5975 C, He carrier, Agilent).

Quasi-in-situ and in-situ XAFS characterization

Ru K-edge X-ray absorption fine structure (XAFS) data collection was performed at the beamline BL14W1 in Shanghai Synchrotron Radiation Facility (SSRF), China. The X-ray was monochromatized by a double-crystal Si (311) monochromator. The storage ring of SSRF was operated at 3.5 GeV with the current of 300 mA.

For in-situ data collection, the sealed homemade canteen-like quartz reactor was filled with 80% H_2/20% CO_2 and *d*-RuNUs catalyst was coated on one of the inner surfaces. Then we placed the region of interest at the path of the incoming hard X-ray (Figure S21). The data started to be recorded as soon as 300 W Xe-lamp began to irradiate.

The data was processed according to the standard procedures using the Demeter program set with a built-in FEFF code. Crystal structural parameters of the Ru foil were used to fit Fourier-transform extended X-ray absorption fine structure (FT-EXAFS) spectra.

The soft X-ray absorption spectroscopy (XAS) data of Ru $M_{2,3}$ edge and C K edge were acquired on the MCD-A and MCD-B Soochow Beamline for Energy Materials at the National Synchrotron Radiation Laboratory (NSRL)

In-situ DRIFTS for photothermal catalytic CO_2 conversion

In-situ DRIFTS measurements were performed at BL01B in the NSRL in Hefei, China. The spectra were collected by using a Bruker IFS 66v Fourier-transform spectrometer equipped with Harrick diffuse reflectance accessory with ZnSe and quartz window. Each spectrum was recorded by averaging 128 scans at a resolution of 2 cm^{-1}. After sample loading, premixed feed gas of 80% H_2/20% CO_2 was introduced into the chamber for background spectra collection. After that, the accessory was heated to 573 K, and a series of spectra were collected during 40 min reaction.

COMSOL Multiphysics computational simulation

The heat transfer was simulated by the solid and fluid heat transfer module using COMSOL Multiphysics software. The physical parameters of density (d-RuNUs: 608 kg m^{-3}, MWCNTs: 270 kg m^{-3}), porosity (0.8), thermal conductivity (d-RuNUs: 100 W m^{-1} K^{-1}, MWCNTs: 1000 W m^{-1} K^{-1}) and thermal capacity (d-RuNUs: 884 J kg^{-1} K^{-1}, MWCNTs: 550 J kg^{-1} K^{-1}) were acquired from built-in archive, open-source data and measurements. For the whole system simulation, the reactor was built by 1:1 scale, and flowing gas was configured as the fully-conversed feed gas with a ratio of 33% CH_4 and 66% g-H_2O. The size of the *d*-RuNUs or *d*-RuNUs/MWCNTs catalyst film was set to be a diameter of 30 mm and thickness of 0.2 mm. After simulation, the temperature scanning vector *r* was configured along the radial direction from the center to the edge of the catalyst film to acquire temperature distribution.

To obtain the temperature distribution along the thickness direction (*h*) of *d*-RuNUs and *d*-RuNUs/MWCNTs catalyst films, a region was extracted randomly to be simulated. The size of the region was 100 nm×100 nm×0.2 mm (the thickness of the catalyst). After simulation, the temperature scanning vector *h* was configured as that from the bottom face center point to the top face center point to acquire temperature.

For the simulation of the extracted microzone of *d*-RuNUs and *d*-RuNUs/MWCNTs, the size of the *d*-RuNUs spheres was configured as 38.2 nm. The *d*-RuNUs spheres were set to be the general heat sources and the power per unit volume is proportional to the total power of the exothermic Sabatier reaction. The size of microzone integration was configured as 125 nm×125 nm×171.3 nm. The reaction time in simulation is 1.5 ps.

Results and Discussion

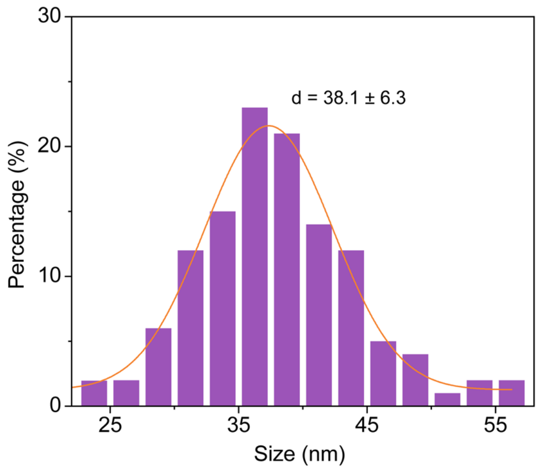

Figure S1. Size distribution data of *d*-RuNUs.

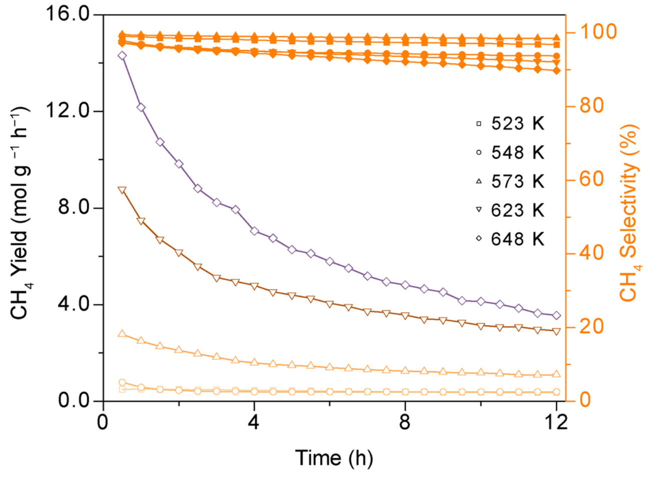

Figure S2. Thermal catalytic Sabatier reaction performance of *d*-RuNUs under different temperatures (GHSV= 450,000 mL g^{-1} h^{-1}).

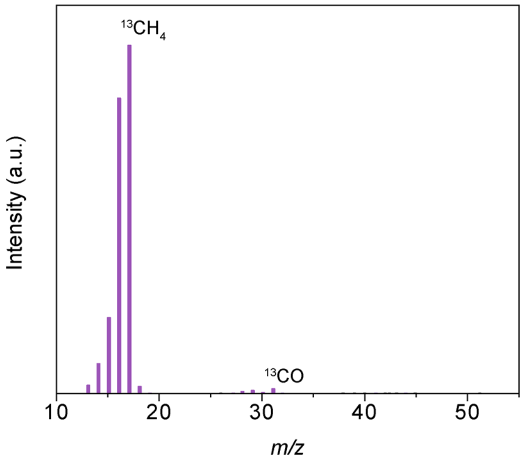

Figure S3. Mass spectra collected after $^{13}CO_2$ isotope-labelling Sabatier reaction.

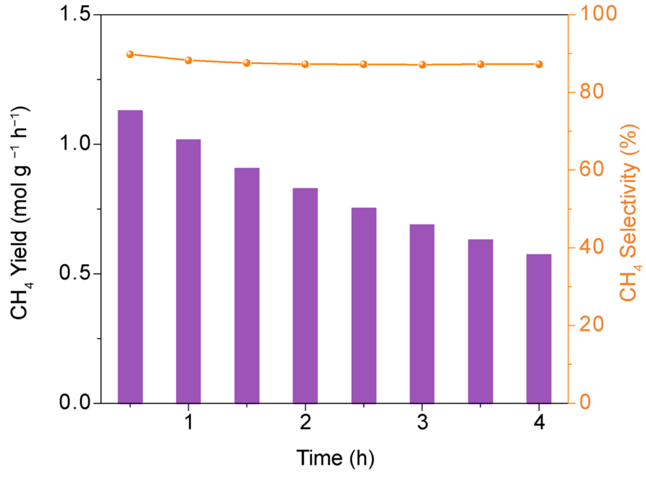

Figure S4. Photothermal catalytic Sabatier reaction performance of regenerated *d*-RuNUs (light intensity 2.0 W cm^{-2}, GHSV= 450,000 mL g^{-1} h^{-1}).

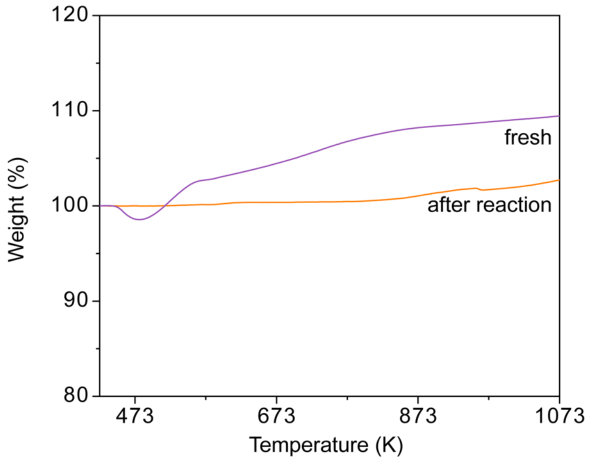

Figure S5. TGA test of fresh *d*-RuNUs and *d*-RuNUs after reaction.

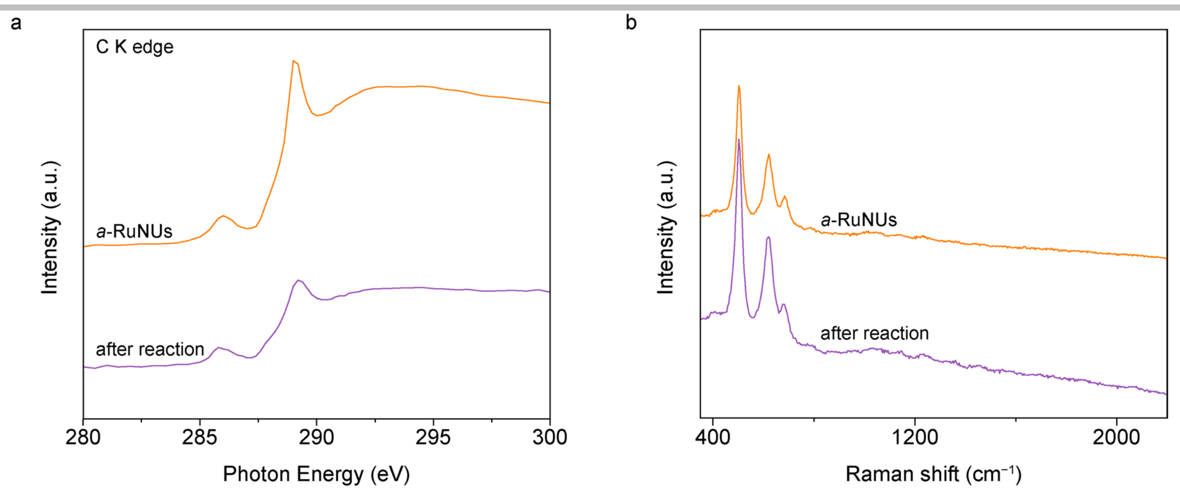

Figure S6. a) C K edge XAS spectra and b) Raman spectra of fresh *d*-RuNUs and *d*-RuNUs after reaction.

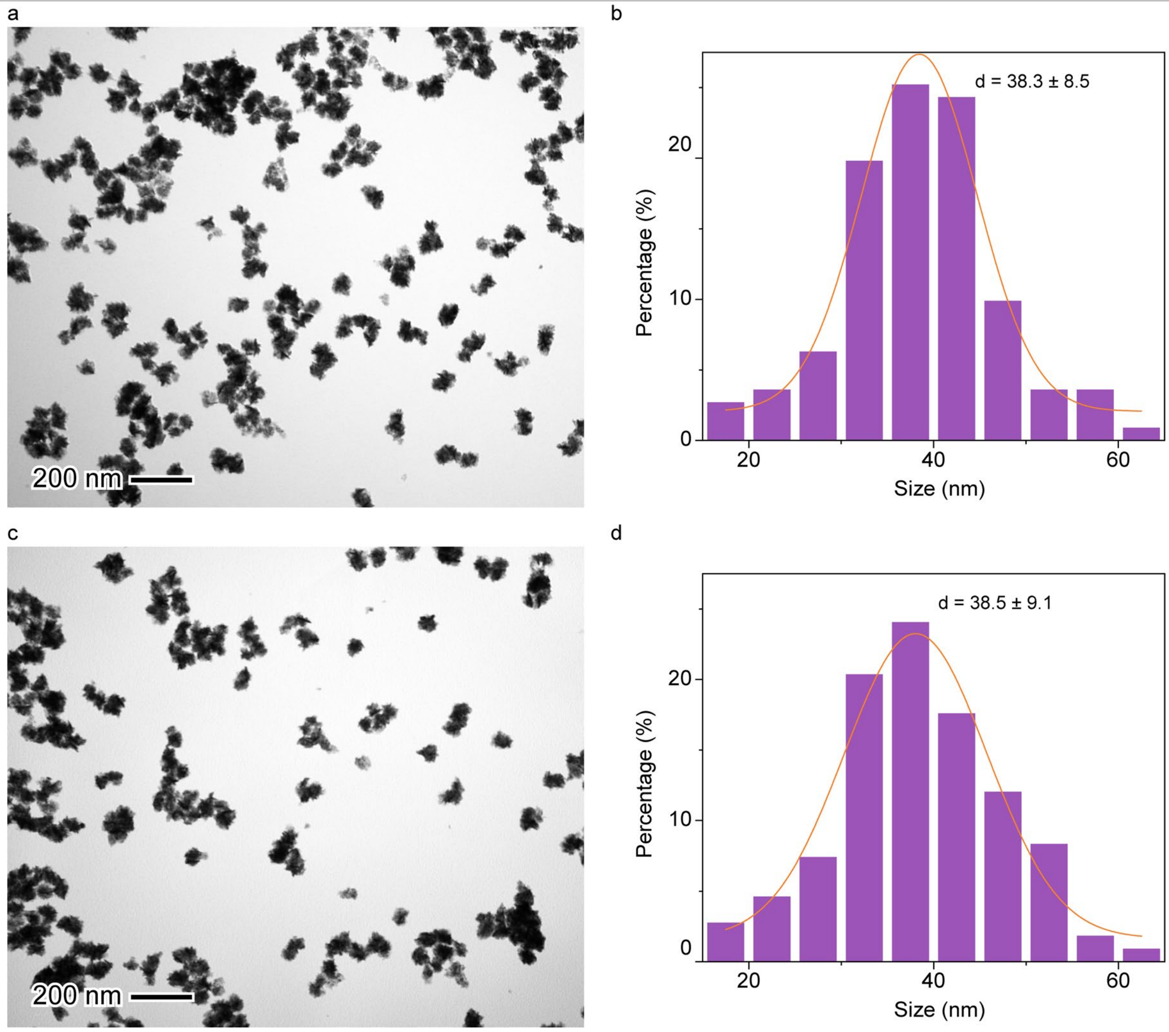

Figure S7. a) TEM image and b) size distribution data of freshly synthesized *d*-RuNUs. c) TEM image and d) size distribution data of *d*-RuNUs after reaction.

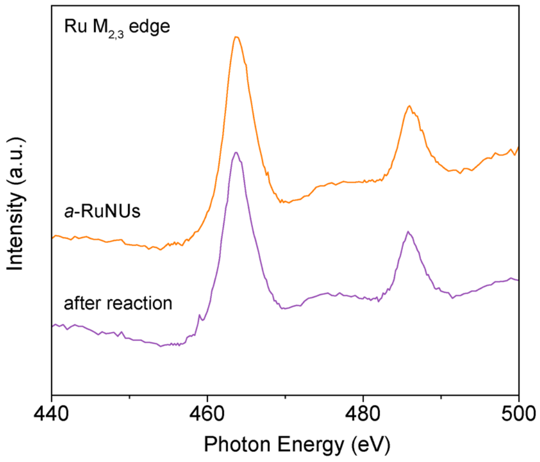

Figure S8. Ru $M_{2,3}$ edge XAS spectra of fresh *d*-RuNUs and *d*-RuNUs after reaction.

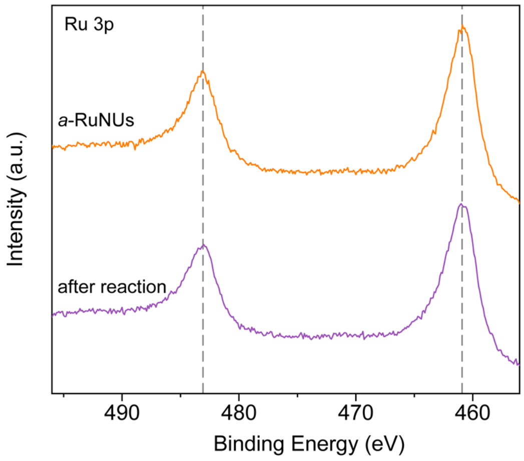

Figure S9. Ru 3p XPS spectra of fresh *d*-RuNUs and *d*-RuNUs after reaction.

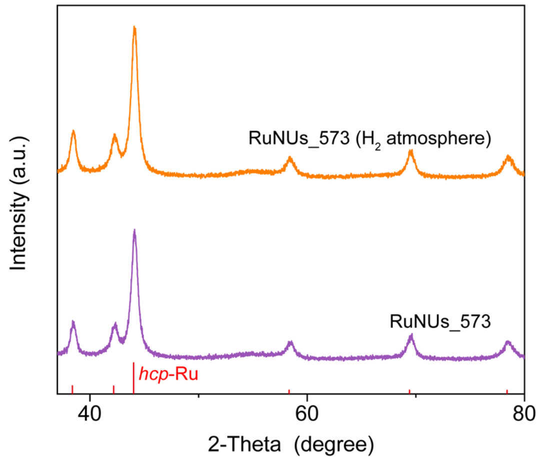

Figure S10. XRD patterns of RuNUs annealed under 573 K in H_2 atmosphere and RuNUs_573.

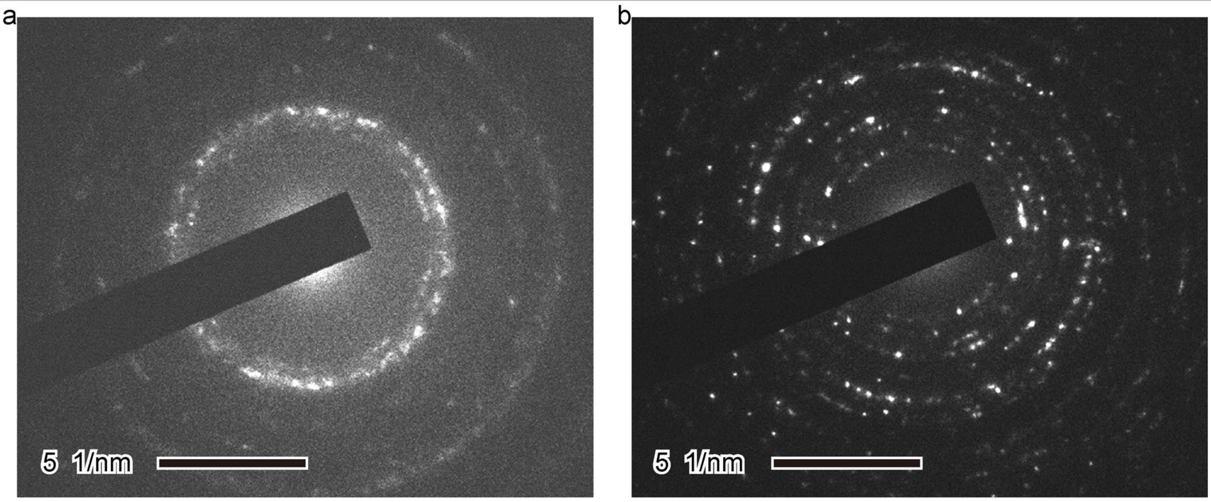

Figure S11. SAED patterns of a) RuNUs_473 and b) RuNUs_773.

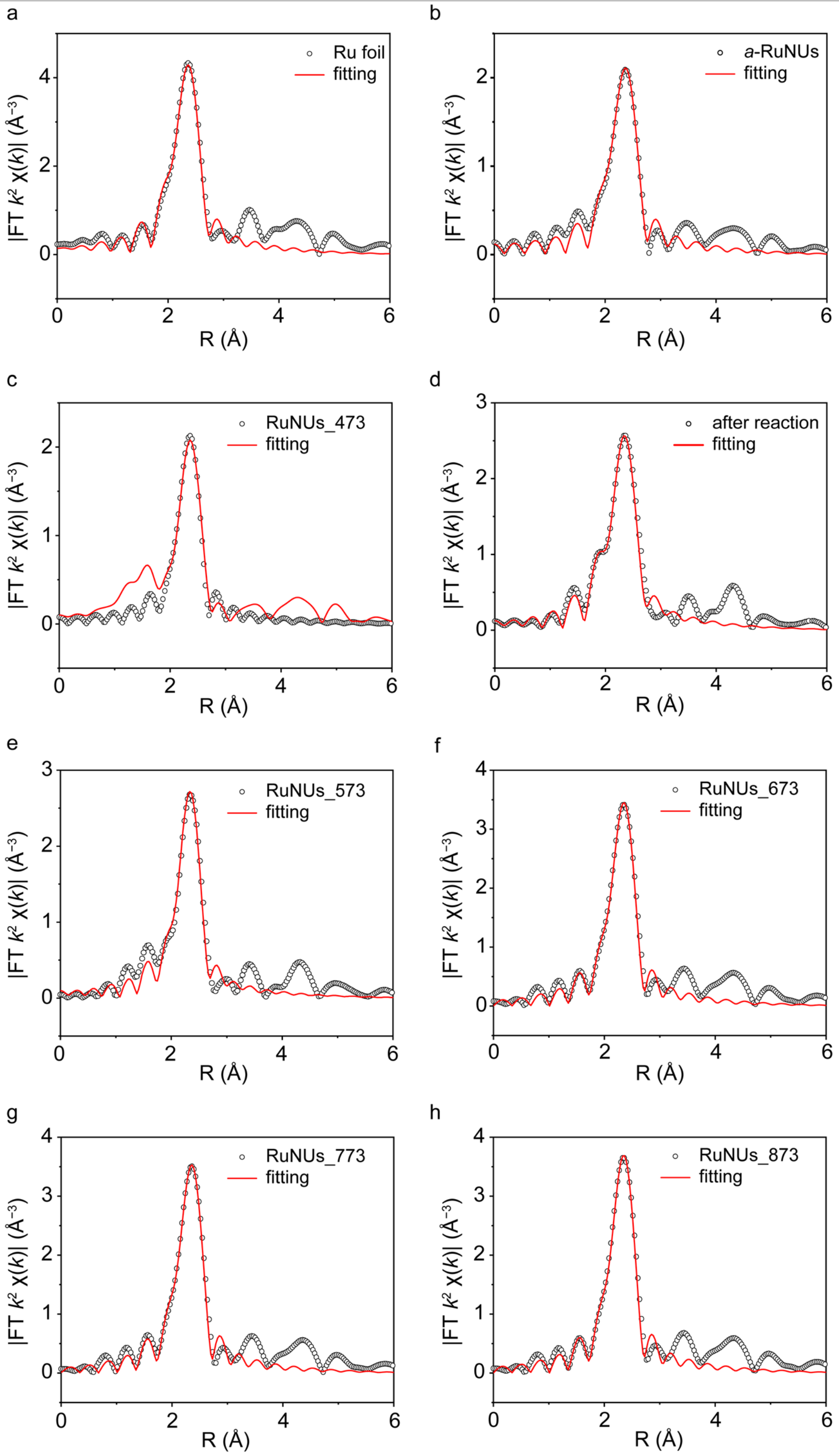

Figure S12. k^2-weighted Ru K edge FT-EXAFS fitting results of a) Ru foil, b) *d*-RuNUs, c) RuNUs_473, d) *d*-RuNUs after reaction, e) RuNUs_573, f) RuNUs_673, g) RuNUs_773 and h) RuNUs_873.

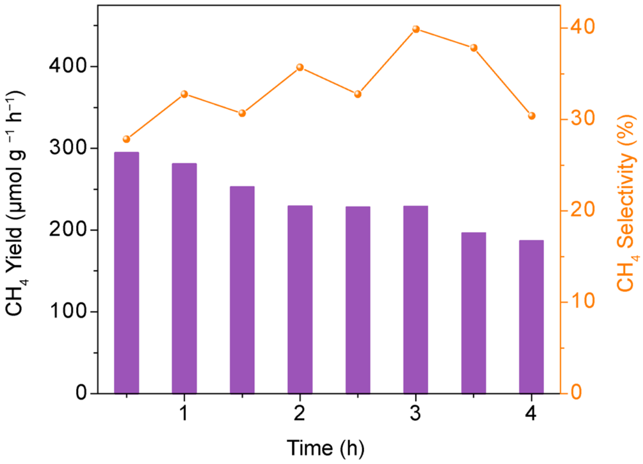

Figure S13. Photothermal catalytic Sabatier reaction performance of RuNUs_873 (light intensity 2.0 W cm^{-2}, GHSV= 450,000 mL g^{-1} h^{-1}).

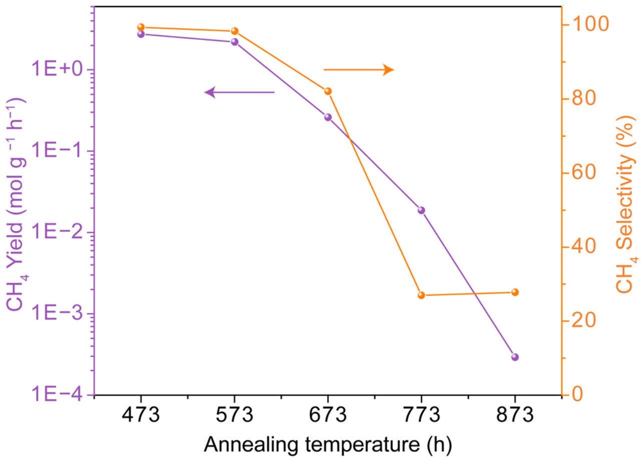

Figure S14. Thermal catalytic Sabatier reaction performance of RuNUs_473, RuNUs_573, RuNUs_673, RuNUs_773 and RuNUs_873 (reaction time: 0.5 h, 573 K, GHSV= 450,000 mL g^{-1} h^{-1}).

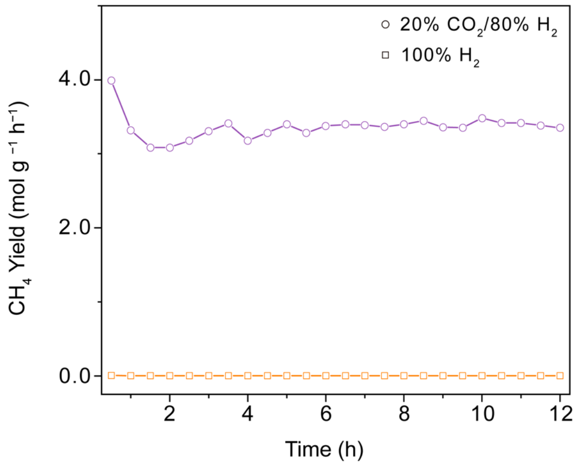

Figure S15. Thermal catalytic methanation performance of *d*-RuNUs/MWCNTs in 100% H_2 and 20% CO_2/80% H_2 atmosphere, indicating the inert nature of MWCNTs (reaction temperature 573 K, GHSV= 450,000 mL g^{-1} h^{-1}).

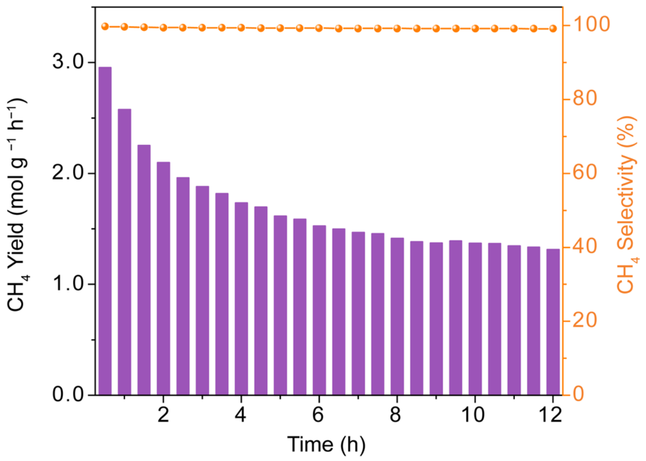

Figure S16. Thermal catalytic Sabatier reaction performance of *d*-RuNUs/graphene (reaction temperature 573 K, GHSV = 450,000 mL g^{-1} h^{-1}).

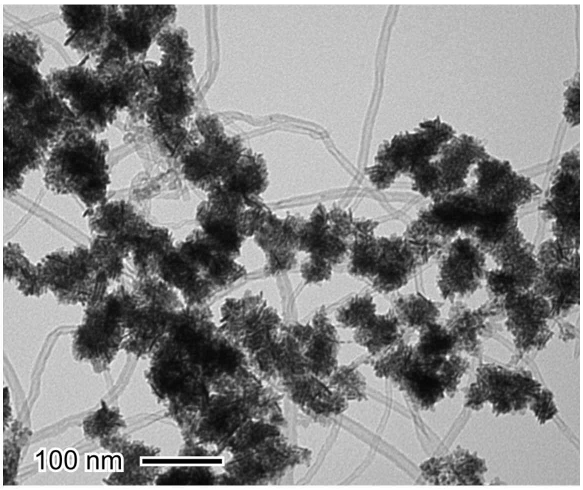

Figure S17. TEM image of *d*-RuNUs/MWCNTs.

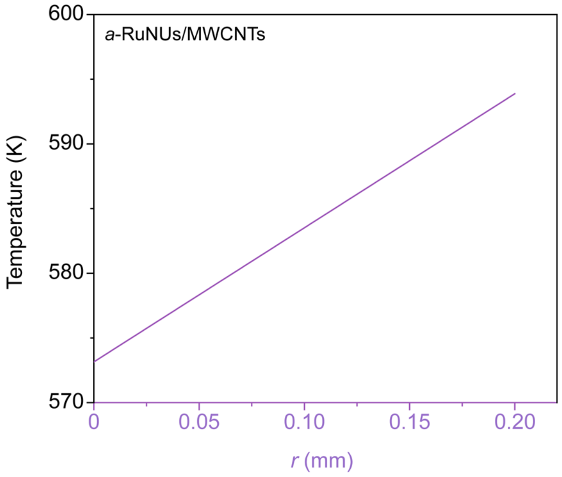

Figure S18. Temperature distribution along *r* vectors of *d*-RuNUs/MWCNTs catalyst film acquired by heat transfer simulation.

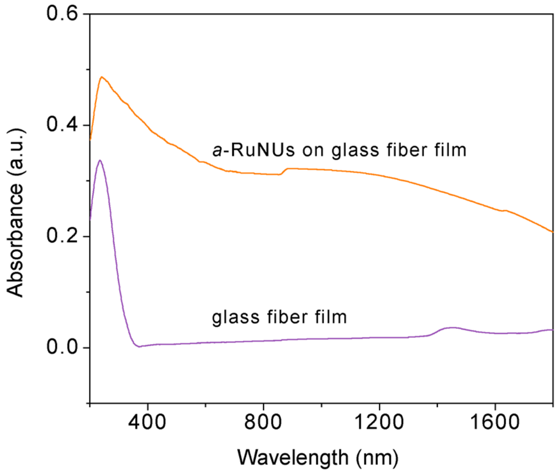

Figure S19. UV-vis spectra of *d*-RuNUs on glass fiber film and individual glass fiber film.

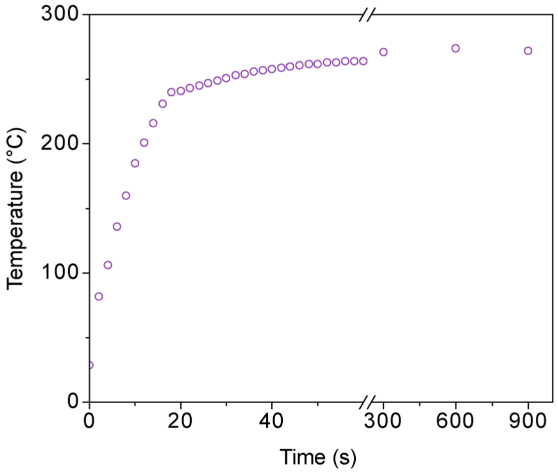

Figure S20. Temperature surveillance data during photothermal catalytic Sabatier reaction under light intensity of 2.0 W cm^{-2} and 80% H_2/20% CO_2.

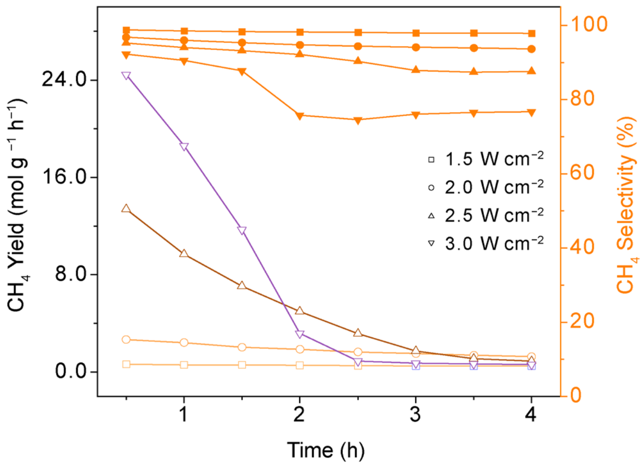

Figure S21. Photothermal catalytic Sabatier reaction performance of *d*-RuNUs under different light intensities (GHSV= 450,000 mL g^{-1} h^{-1}).

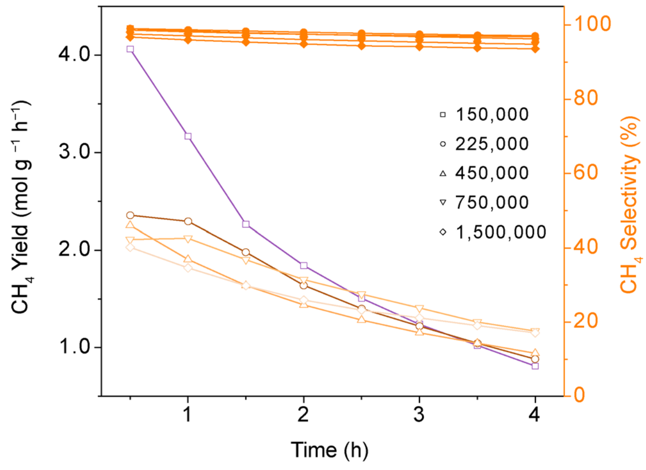

Figure S22. Photothermal catalytic Sabatier reaction performance of *d*-RuNUs under different GHSV (light intensity 2.0 W cm^{-2}).

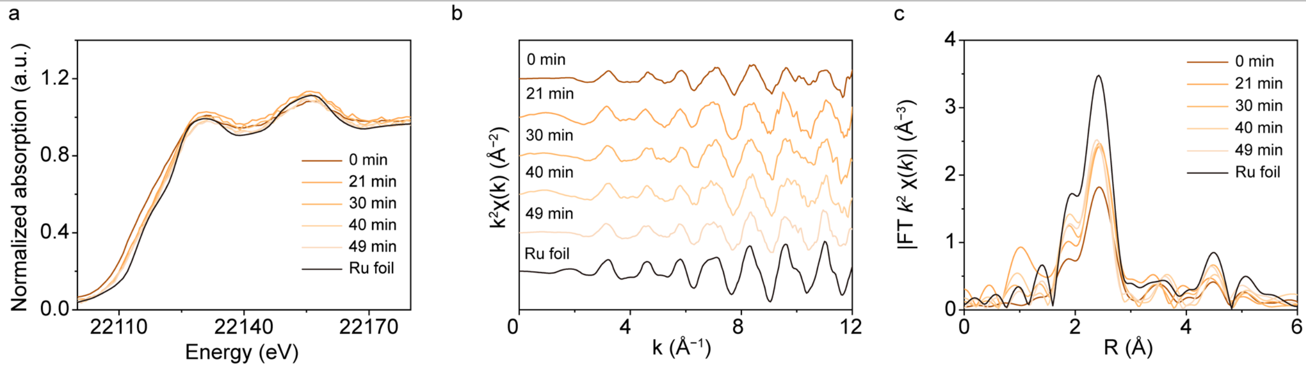

Figure S23. In-situ characterization of Ru K edge a) XANES, b) k^2-weighted EXAFS spectra and c) k^2-weighted FT-EXAFS spectra.

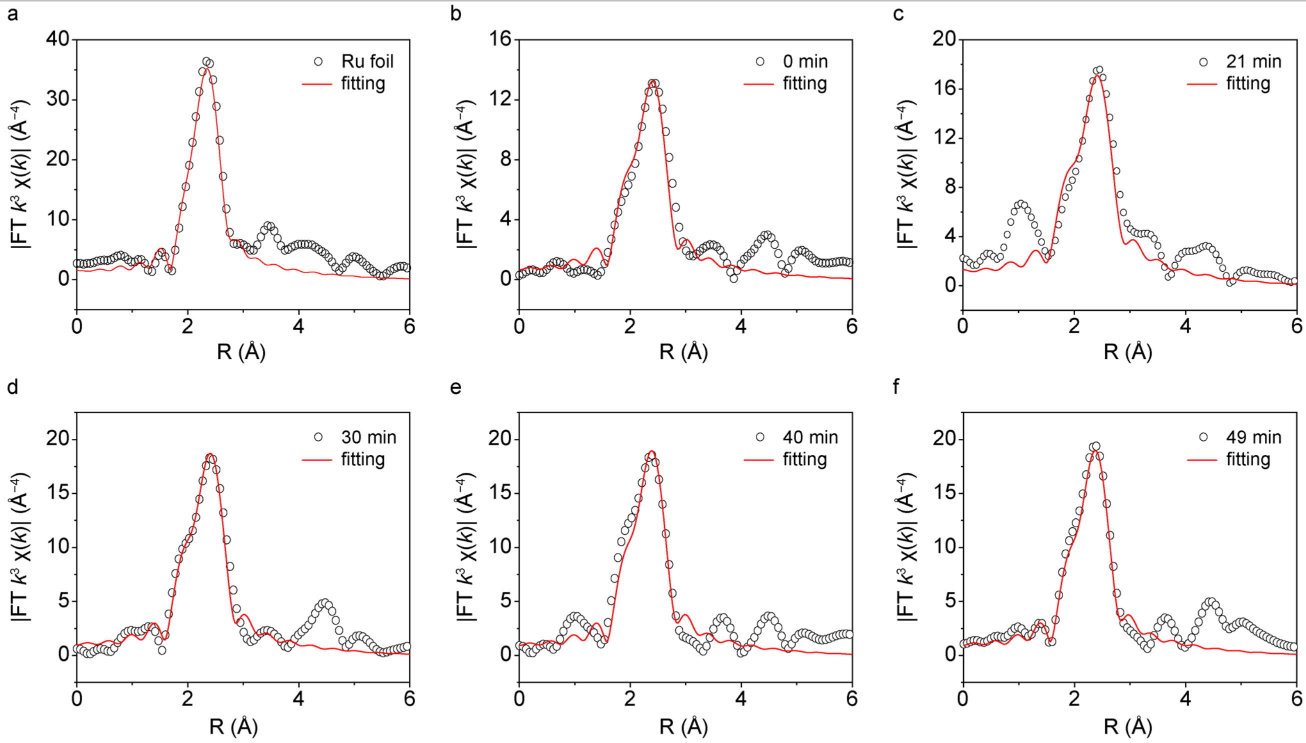

Figure S24. Ru K edge k^3-weighted FT-EXAFS fitting results of a) Ru foil, and *d*-RuNUs samples during in-situ experiment for b) 0 min, c) 21 min, d) 30 min, e) 40 min and f) 49 min.

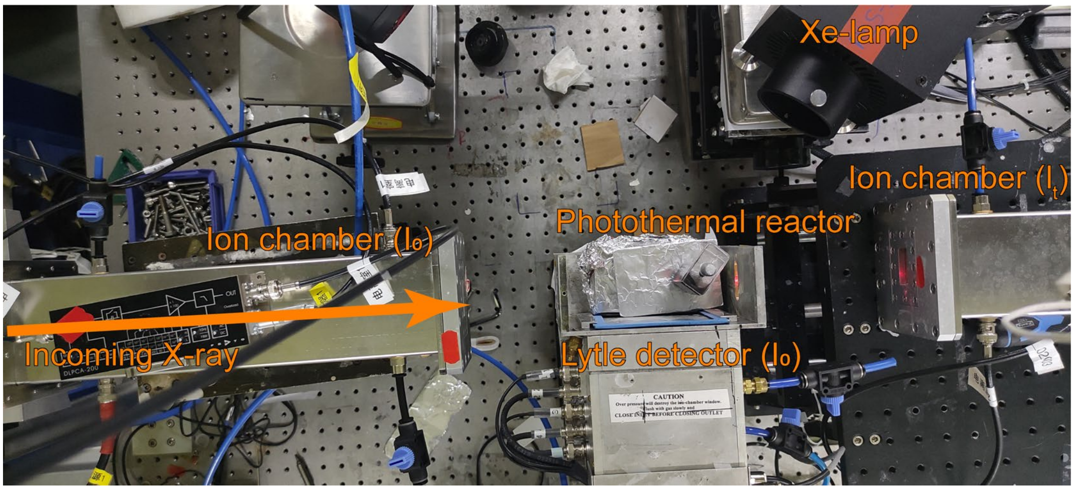

Figure S25. Layout of in-situ XAFS characterization.

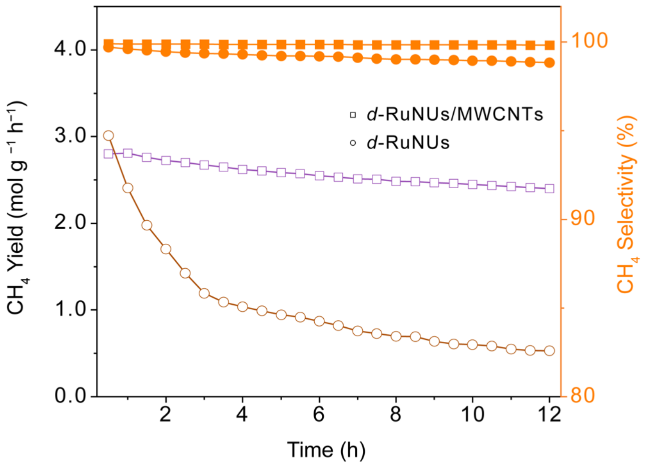

Figure S26. Photothermal catalytic Sabatier reaction performance of *d*-RuNUs/MWCNTs and *d*-RuNUs (light intensity 2.0 W cm^{-2}, GHSV= 450,000 mL g^{-1} h^{-1}).

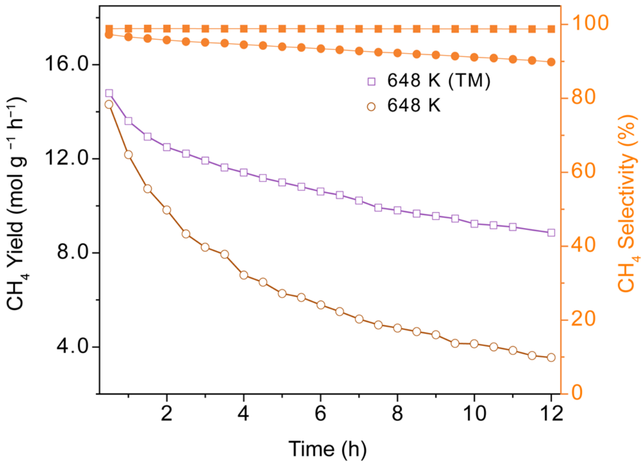

Figure S27. Thermal catalytic Sabatier reaction performance of *d*-RuNUs and *d*-RuNUs when employing thermal management method (TM) under 648 K (reaction temperature 573 K, GHSV= 450,000 mL g^{-1} h^{-1}).

Table S1. Fitting results of quasi-in-situ Ru K edge EXAFS Data.[a]

Sample	Scattering Path	CN	R(Å)	ΔE_0(eV)	σ^2 (Å)
Ru foil	Ru-Ru	12 (fixed)	2.67 +/- 0.06	6.94 +/- 0.96	0.00248
fresh	Ru-Ru	6.63 +/- 0.43	2.67 +/- 0.05	6.67 +/- 1.39	0.00334
after reaction	Ru-Ru	8.76 +/- 0.41	2.67 +/- 0.06	7.96 +/- 0.93	0.00334
RuNUs_473	Ru-Ru	6.23 +/- 0.67	2.67 +/- 0.06	6.95 +/- 2.29	0.00334
RuNUs_573	Ru-Ru	7.64 +/- 0.49	2.67 +/- 0.06	8.17 +/- 1.35	0.00335
RuNUs_673	Ru-Ru	9.92 +/- 0.42	2.67 +/- 0.06	7.65 +/- 0.88	0.00332
RuNUs_773	Ru-Ru	10.17 +/- 0.44	2.67 +/- 0.06	6.92 +/- 0.89	0.00335
RuNUs_873	Ru-Ru	10.65 +/- 0.42	2.67 +/- 0.06	7.76 +/- 0.86	0.00334

[a] CN, the coordination numbers; R, the bonding distance; σ^2, the Debye-Waller factor; S_0^2 was set to 0.68.

Table S2. Fitting results of in-situ Ru K edge EXAFS Data.[a]

Sample	Scattering Path	CN	R(Å)	ΔE_0(eV)	σ^2 (Å)
Ru foil	Ru-Ru	12 (fixed)	2.67 +/- 0.06	6.94 +/- 0.96	0.00266
0 min	Ru-Ru	6.15 +/- 0.87	2.65 +/- 0.08	0.15 +/- 2.34	0.00449
21 min	Ru-Ru	8.44 +/- 0.80	2.66 +/- 0.06	1.80 +/- 1.62	0.00364
30 min	Ru-Ru	8.66 +/- 0.83	2.66 +/- 0.07	0.01 +/- 1.56	0.00440
40 min	Ru-Ru	8.67 +/- 0.71	2.65 +/- 0.08	1.11 +/- 1.33	0.00453
49 min	Ru-Ru	8.69 +/- 0.43	2.65 +/- 0.08	2.70 +/- 0.80	0.00450

[a] CN, the coordination number; R, the bonding distance; σ^2, the Debye-Waller factor; S_0^2 was set to 0.80.

Table S3. Recent representative works of catalytic Sabatier reaction.

Catalysts	Thermal catalytic reaction temperature (K)	Photothermal catalytic reaction temperature (K)	Photothermal catalysis light source and intensity (W cm^{-2})	Gas hour space velocity (mL g^{-1} h^{-1})	Selectivity (%) and yield of CH_4 (mol g^{-1} h^{-1})	Reaction time without deactivating (h)	Reference
d-RuNUs in thermal catalysis	573	-	-	450,000	99.8; 3.2	12	This work
d-RuNUs in photothermal catalysis	-	545 (measured)	300W Xe lamp; 2.0	450,000	Up to 96.9; 2.7	-	This work
Ni/CeO_2-La-600	598	-	-	30,000	99%; 0.2	100	[1]
NiMg-MOF-74	623	-	-	48,000	97%; 0.2	12	[2]
Sr Promoted Ni/W−Zr	623	-	-	18,000	90%; 0.1	5	[3]
15% Ni/MCN	723	-	-	120,000	100%; 4.5	9	[4]
Ni/CeO_2-nanorods	523	-	-	20,000	97.8%; 0.2	150	[5]
Ni-$BaTiO_3$	-	270	300W Xe lamp; 0.293	- (batch reactor)	Nearly 100%; 0.1	2.3	[6]
Ru/$MnCo_2O_4$	-	230	420-780 nm; 1.25	24,000	96%; 0.07	12	[7]
MOF-derived defective Ni/TiO_2	-	330	IR light; 1.23	15,000	>99%; 0.02	48	[8]
Ru@$Ni_2V_2O_7$	-	350	300W Xe lamp; 2.0	- (batch reactor)	95%; 0.1	10	[9]
Ni-TiO_2		325	300 W Xe lamp; 1.2	24000	99%; 0.1	4	[10]

References

[1] T. Zhang, W. Wang, F. Gu, W. Xu, J. Zhang, Z. Li, T. Zhu, G. Xu, Z. Zhong, F. Su, *Applied Catalysis B: Environmental* **2022**, *312*, 121385.

[2] T. Zurrer, K. Wong, J. Horlyck, E. C. Lovell, J. Wright, N. M. Bedford, Z. Han, K. Liang, J. Scott, R. Amal, *Advanced Functional Materials* **2021**, *31*, 2007624.

[3] A. S. Al-Fatesh, M.-N. Kaydouh, H. Ahmed, A. A. Ibrahim, M. F. Alotibi, A. I. Osman, N. El Hassan, *Langmuir* **2023**, *39*, 17723-17732.

[4] Z. Refaat, M. E. Saied, A. O. A. E. Naga, S. A. Shaban, H. B. Hassan, M. R. Shehata, F. Y. E. Kady, *Scientific Reports* **2023**, *13*, 4855.

[5] Y. Xie, J. Chen, X. Wu, J. Wen, R. Zhao, Z. Li, G. Tian, Q. Zhang, P. Ning, J. Hao, *ACS Catalysis* **2022**, *12*, 10587-10602.

[6] D. Mateo, N. Morlanes, P. Maity, G. Shterk, O. F. Mohammed, J. Gascon, *Advanced Functional Materials* **2021**, *31*, 2008244.

[7] C. Guo, Y. Tang, Z. Yang, T. Zhao, J. Liu, Y. Zhao, F. Wang, *ACS Nano* **2023**, *17*, 23761-23771.

[8] Q. Li, Y. Gao, M. Zhang, H. Gao, J. Chen, H. Jia, *Applied Catalysis B: Environmental* **2022**, *303*, 120905.

[9] Y. Chen, Y. Zhang, G. Fan, L. Song, G. Jia, H. Huang, S. Ouyang, J. Ye, Z. Li, Z. Zou, *Joule* **2021**, *5*, 3235-3251.

[10] P. Li, S. Zhang, Z. Xiao, H. Zhang, F. Ye, J. Gu, J. Wang, G. Li, D. Wang, *Fuel* **2024**, *357*, 129817.

Author Contributions

D.D. and D.W. contributed to this work equally.
